# Experimental Investigation and Statistical Evaluation of Optimized Cutting Process Parameters and Cutting Conditions to Minimize Cutting Forces and Shape Deviations in Al6026-T9

**DOI:** 10.3390/ma13194327

**Published:** 2020-09-29

**Authors:** Muhammad Abas, Bashir Salah, Qazi Salman Khalid, Iftikhar Hussain, Abdur Rehman Babar, Rashid Nawaz, Razaullah Khan, Waqas Saleem

**Affiliations:** 1 Department of Industrial Engineering, University of Engineering and Technology, Peshawar 25120, Pakistan; qazisalman@uetpeshawar.edu.pk (Q.S.K.); iftikhar@uetpeshawar.edu.pk (I.H.); abdurrehman@uetpeshawar.edu.pk (A.R.B.); rnawaz@uetpeshawar.edu.pk (R.N.); 2 Industrial Engineering Department, College of Engineering, King Saud University, P.O. Box 800, Riyadh 11421, Saudi Arabia; bsalah@ksu.edu.sa; 3 Department of Mechanical Engineering Technology, University of Technology, Nowshera 24100, Pakistan; 4 Department of Mechanical and Manufacturing Engineering, Institute of Technology, F91 YW50 Sligo, Ireland; waqas95@yahoo.com

**Keywords:** aluminum alloy, minimum quantity lubricant (MQL), cutting forces, shape deviations, taguchi orthogonal array design, analysis of variance (ANOVA), multi-objective optimization based on ratio analysis (MOORA), criteria importance through inter-criteria correlation (CRITIC)

## Abstract

Precise, economical and sustainable cutting operations are highly desirable in the advanced manufacturing environment. For this aim, the present study investigated the influence of cutting parameters (i.e., the cutting speed (*c*), feed rate (*f*), depth of cut (*d*) and positive rake angle (*p*)) and sustainable cutting conditions (dry and minimum quantity lubricant (MQL)) on cutting forces (i.e., feed force (F_f_), tangential forces (F_t_), radial force (F_r_) and resultant cutting forces (F_c_) and shape deviations (i.e., circularity and cylindricity) of a 6026-T9 aluminum alloy. The type of lubricant and insert used are virgin olive oil and uncoated tungsten carbide tool. Turning experiments were performed on a TAKISAWA TC-1 CNC lathe machine and cutting forces were measured with the help of a Kistler 9257B dynamometer. Shape deviations were evaluated by means of a Tesa Micro-Hite 3D DCC 474 coordinate measuring machine (CMM). Experimental runs were planned based on Taguchi mixture orthogonal array design L16. Analysis of variance (ANOVA) was performed to study the statistical significance of cutting parameters. Taguchi based signal to noise (S/N) ratios are applied for optimization of single response, while for optimization of multiple responses Taguchi based signal to noise (S/N) ratios coupled with multi-objective optimization on the basis of ratio analysis (MOORA) and criteria importance through inter-criteria correlation (CRITIC) are employed. ANOVA results revealed that feed rate, followed by a depth of cut, are the most influencing and contributing factors for all components of cutting forces (F_f_, F_t_, F_r_, and F_c_) and shape deviations (circularity and cylindricity). The optimized cutting parameters obtained for multi responses are *c* = 600 m/min, *f* = 0.1 mm/rev, *d* = 1 mm and *p* = 25°, while for cutting conditions, MQL is optimal.

## 1. Introduction

Turning of aluminum alloys have gained paramount significance in automobile and aerospace advanced manufacturing. This is due to its high strength to weight ratio [1,2,3]. Turning is an important machining process for the cutting of round objects to the desired shape and size. It has the advantages of producing a good quality product, having a lower lead time, customer satisfaction and being economical [1]. However, while machining such alloys, sustainability of the cutting operation to meet the surface finish, dimensional precision, lower cutting forces, with economic power consumption is highly desirous [1,2]. From this perspective, the appropriate selection of cutting parameters, especially feed rate, cutting speed, and depth of cut are essential to achieve these objectives [1,2]. Further, machining can be performed in dry conditions [3,4], near dry conditions (minimum quantity lubricant, MQL) [3,4], flooded coolant [3,4], cryogenic coolant [3,4], or with nanofluids [5,6]. However, the growing concept of green/sustainable manufacturing has shifted the paradigm of manufacturers to environmentally friendly cutting fluids [3,4]. Dry and MQL environment are considered sustainable in machining processes [5]. The advantages of MQL are to reduce cutting forces/cutting power and temperature at the tooltip, improve the tool life, enhance dimensional accuracy, and improve the surface quality of machined parts [3,4,5]. The most common eco-friendly cutting fluids are vegetable oil [3,4,6,7], nano cutting fluids, and ester [3,4,6,7] due to the less poisonous effects and biodegradability as compared to petroleum-based mineral oil [5,6,7].

## 2. Literature Review

In the presented research study, a comprehensive literature review of the turning operation of aluminum alloys has been carried out under dry, MQL, and flooded conditions. For example, Patel et al. [8] have analyzed and optimized the surface quality characteristics (i.e., circularity and cylindricity errors) and material removal rate in the dry turning of a 7075 aluminum alloy. The analysis of variance (ANOVA) showed that cutting speed is the most influencing factor in all responses. They reported that an increase in cut depth and feed rate instigate a negative impact on the surface quality, circularity, and cylindricity error, while creating a positive effect on the material removal rate. Surface quality improves with an increase in the nose radius improves while having a non-linear relationship with circularity and cylindricity error. They also optimized cutting parameters using JAYA coupled with principal component analysis (PCA). Ajay and Vinoth [9] optimized the turning cutting parameters of 6061 aluminum alloy using high-speed steel (HSS) insert under dry condition. The ANOVA results revealed that cutting speed is the most significant factor for surface roughness, while for temperature and resultant cutting forces, cutting speed, feed rate, and depth of cut are the most significant contributing factors. The study also showed that cutting forces increase with an increase in feed rate and depth of cut. A similar study was completed by Javidikia et al. [10]. They studied the impact of tool geometry and cutting conditions in turning the aluminum alloy 6061-T6 under a dry condition with uncoated carbide insert. Their results indicated that machining forces decrease with an increase in cutting speed; however, they increases with an increase in cutting edge radius. The temperature at the tooltip interface increases with an increase in cutting speed and decreases with an increase in rake angle from negative to positive values. Likewise, a decrease in the cutting forces was observed by increasing the rake angle from negative to positive values. An increase in feed rate increases feed forces; however, it reduces cutting forces. Kannan et al. [11] studied the machinability of the aluminum matrix Al 7075/BN/Al_2_O_3_ under MQL and dry conditions using a grade K313 (WC/Co fine-grain grade) cutting tool. They investigated that machining under a MQL environment reduces the cutting force, tool wear, and improves the quality of the machined surface compared to the dry environment. Additionally, low cutting forces are observed at high cutting speed and low feed rate. An investigation on tool wear in the turning of an Al/SiCp (based on aluminum alloy 2024) composite under cooling and lubrication conditions was conducted by Duan et al. [12]. The insert used was a polycrystalline diamond (PCD) tool. Their study showed that the type of flank wear, abrasive wear, and tool breakage could be controlled up to a greater extent under MQL (a mixture of oil and gas) and liquid nitrogen (LN_2_). Kouam et al. [13] studied the effect of MQL conditions on the machining of a 7075-T6 aluminum alloy. The cutting tool insert used was a carbide (DNGP-432 KC5410, Kennametal, Latrobe, PA, USA) with Titanium diboride (TiB_2_) coating, while the coolant was Mecagreen 550 lubricant coolant mixed with 15% water at 3 and 1.75 mL/min flow rate. The effect of MQL in the machining of a 6082 aluminum alloy was studied by Yigit [14]. The diamond-coated carbide was used as insert, while the lubricant was commercial oil Rocol A208A plus, applied at 50 mL/h and 100 mL/h. His study concluded that surface roughness, dimensional accuracy, and cutting forces improved due to the reduction in wear at the tooltip under MQL conditions. Islam [15] analyzed surface roughness and dimensional accuracy (diameter error and circularity) in the turning of an aluminum 6061, mild steel 1030, and alloy steel 4340. The insert used was a square-shaped insert with enriched cobalt coating (chemical vapor deposition (CVD) titanium nitride (TiN) – titanium carbonitride (TiCN) – aluminium oxide (Al_2_O_3_) –TiN) manufactured by Stellram, La Vergne, TN, USA. The coolant was 2010 Coolube, a vegetable-based metal cutting lubricant, and sprayed in the form of mist at 1.667 × 10^−5^ L/s flow rate. The ANOVA results revealed that the work material and coolant methods (MQL) have a significant effect on the dimensional accuracy and the least effect on surface roughness.

Jafarian et al. [16] optimized the multiple responses including resultant cutting forces, insert wear, and surface roughness, in aluminum alloy turning using an integrated approach of artificial neural network, genetic algorithm (GA) and particle swarm optimization (PSO). The optimized results indicated that the proposed methodology is effective in predicting optimal cutting parameters. Agustina et al. [17] analyzed the cutting forces in the turning of unified numbering system (UNS) A97075 aluminum alloys under dry conditions with two types of inserts i.e., DCMT11T304-F2 and DCMT11T308-F2 manufactured by SECO, Aljunied, Singapore. The most significant parameters that affect the cutting forces are feed rate, followed by depth of cut and tool type. Sreejith [18] studied the performance of the machining of a 6061 aluminum alloy with dry, MQL, and flooded lubricant conditions. The diamond-coated carbide was used as insert, while the lubricant was commercial oil BP Microtrend 231 L. The flow rate of MQL was 50 and 100 mL/h. The results showed that machining under MQL conditions provides comparable results to flooded lubricant conditions. The thermal softening of chips during the machining of aluminum alloys affects the surface quality, cutting forces, and tooltip. However, with the application of MQL, it can be reduced to a greater extent. Reis and Abrao [19] examined the machinability of the 6351-T6 aluminum alloy under dry turning conditions. The inserts used were cemented carbide, diamond coated carbide, and polycrystalline diamond (PCD). The results revealed that the PCD tool performed better compared to other tools. Further, cutting forces increase with an increase in feed rate and depth of cut, however, they decrease with an increase in cutting speed. For the PCD tool, the dominant force observed was the radial force, while the tangential and axial forces were lowest.

The Taguchi based signal to noise (S/N) ratios method is an experimental design technique that is suitable for the optimization of a single response variable [20]. In the presented study, we have focused multi-responses, and consequently, in order to deal with such a problem, the multi-objective optimization based on ratio analysis (MOORA) method is selected. This method was proposed by Brauers [21] and can successfully deal with the complex decision-making process in the manufacturing environment [22]. It allows us to simultaneously optimize the responses, whether their objective function is conflicting (including both maximization and minimization terms) or the same (either maximization or minimization) [23]. According to Yusuf and Sebla [23], MOORA is robust and straightforward compared to other multi-criteria decision-making methods (MCDM) such as technique for order of preference by similarity to ideal solution (TOPSIS), Viekriterijumsko Kompromisno Rangiranje (VIKOR), grey relational analysis and weighted principal components as these methods are complex and difficult to apply in reality. Finding the weights of criteria in MCDM is important [24]. Various methods for weight determination are proposed and are classified into objective and subjective methods. In objective weight methods, the weights of criteria (responses) are measured based on the available data without intervention of an expert’s opinion [24]. The well-known techniques are entropy [25], standard deviation [26], criteria importance through inter-criteria correlation (CRITIC) [27], and the maximizing deviation method [28]. In contrary, the subjective methods involve the expert’s opinion [24]. The most common methods are the pairwise comparison method, namely the analytical hierarchy process (AHP) [29], the swing weighting method [30], the ranking method [31], and the simple multi-attribute rating technique (SMART) [32]. Weight determination using criteria importance through inter-criteria correlation (CRITIC) is effective, because it accounts for both conflict and contrast in weight determination [27]. However the other methods such as entropy, standard deviation, mean weight and maximizing deviation method don’t take into account such information in weight determination [24]. The subjective weight methods, such as AHP, however include expert opinions but do not incorporate the uncertainty and ambiguity of the human mind [24]. Singarave et al., [33] optimized the turning operation of EN25 steel using MOORA coupled with entropy. Pathapalli et al., [34] optimized the machining parameters of an Al-6063 composite using weighted aggregated sum product assessment (WASPAS) and MOORA. They concluded that both methods yielded similar results. Majumder and Saha [35] concluded that MOORA coupled with PCA performed better compared to TOPSIS coupled with PCA in optimizing the turning of ASTM A588 mild steel. Akkaya et al. [36] coupled MOORA with AHP to solve the problem for the industrial engineers in selecting which sector to work in, in the future.

The literature review presented herein shows that a limited number of research publications are available on the machining of aluminum alloys and their composite matrix under dry and MQL environments. Hence, it can be assumed that the presented research demonstrates the first comprehensive attempt to analyze and optimize the cutting forces and shape deviations of the aluminum alloy 6026-T9 under both dry and MQL environments using vegetable oil (namely olive oil). A study performed by Abas et al., [37] on a similar type of material, focuses only on the optimization of surface roughness profile, material removal rate, and tool life under MQL and dry conditions. They concluded that under the MQL environment, the machining of such alloys performs better compared to the dry environment. However, the effect of cutting parameters on the component of cutting forces and shape deviations were not considered in their study. Therefore, in the present research, the Taguchi signal to noise ratio and analysis of variance (ANOVA) are applied to optimize the individual responses in order to achieve this aim. Further, the effect of cutting parameters on performance factors (responses) are studied by using the main effect plots. For multi-response optimization, an integrated approach is implemented by utilizing the Taguchi signal to noise ratio integrated with MOORA and CRITIC.

## 3. Materials and Methods

### 3.1. Experimental Environment

**Figure 1:**
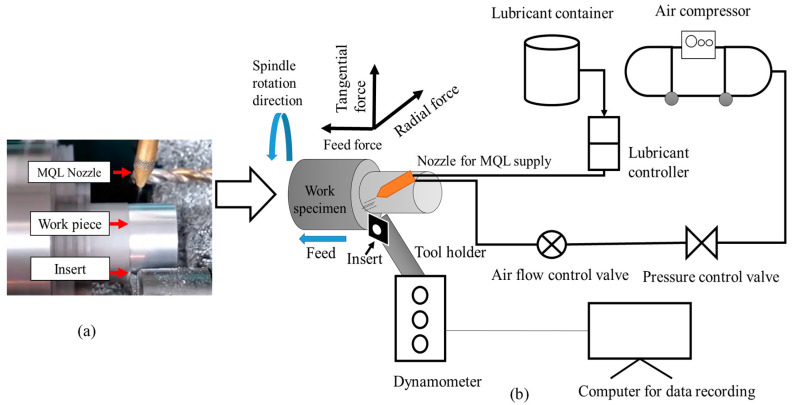
(**a**) Machining of aluminum alloy specimen (**b**) Schematic of an experimental setup. MQL: minimum quantity lubricant.

Turning experiments were performed on a TAKISAWA TC-1 CNC lathe machine (spindle speed 40–4500 rpm, spindle drive motor 5.5 kW, Takisawa, Okayama, Japan) using 6026-T9 aluminum alloy samples (60 mm length and 40 mm in diameter). The uncoated tungsten carbide tool is used as insert having a nose radius of 0.2 mm and a clearance angle of 7°. For each experiment, machining was completed up to a length of 2 cm using a new cutting tool. The machining is performed under dry and minimum quantity lubricant (MQL) conditions. Figure 1a shows the schematic of the coolant supply set up. The type of lubricant used is a vegetable oil (i.e., virgin olive oil) with a viscosity of 84 cp at 20 °C, specific gravity of 0.911 at 20 °C, and a boiling point of 700 °C. The lubricant was supplied at a flow rate of 150 mL/h and 5 bar pressure. The schematic of the experimental setup is shown in Figure 1b.

**Figure 2:**
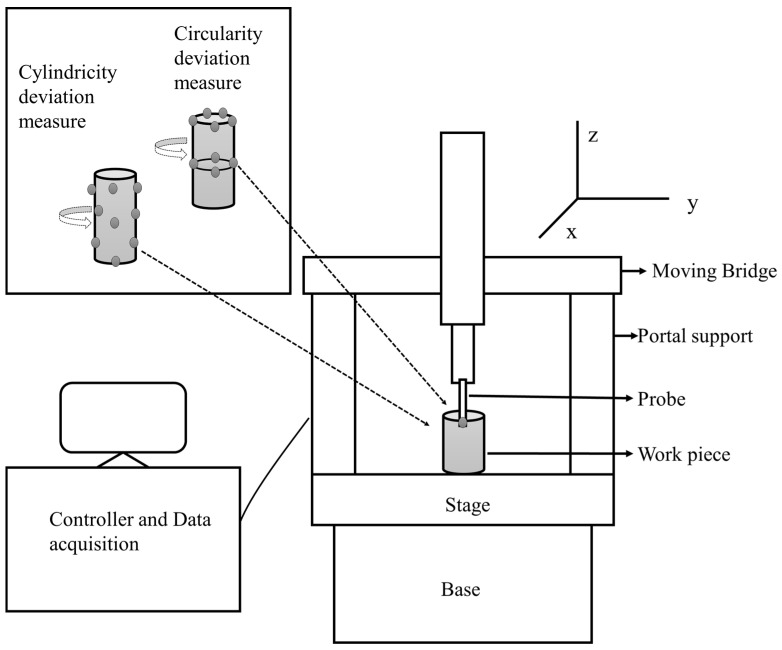
Schematic of a coordinate measuring machine.

The cutting parameters considered are cutting speed, feed rate, depth of cut, and positive rake. The Taguchi orthogonal array mixture design is adopted in this study for experimental runs. Four levels are set for each cutting process parameter (as continuous factors), and two levels are set for cutting conditions (as a categorical factor). These are presented in Table 1. Based on these levels, a total of 16 experimental runs are planned, as tabulated in Table 2. The levels are set based on the literature review and recommendation by the tool manufacturer. The responses to be optimized are components of cutting forces and shape deviations of machined cylindrical bars. The cutting forces i.e., feed force (F_f_), tangential forces (F_t_), radial force (F_r_) and resultant cutting forces (F_c_) are experimentally calculated by using a Kistler 9257B dynamometer (Kistler, Winterthur, Switzerland) with the Kistler multichannel charge amplifier type 5070A (Kistler, Winterthur, Switzerland). Shape deviations are measured in terms of circularity or roundness (mm) based on ISO 6318 standard [38] and cylindricity (mm) deviation based on ISO 12180 standard [39] by using Tesa Micro-Hite 3D DCC 474 a coordinate measuring machine (Tesa SA, Renens, Switzerland) (CMM). The schematic of CMM is shown in Figure 2. The desired measured values of cylindricity (C_y_) and circularity (C_r_) for an experimental run are tabulated in Table 2. Circularity and cylindricity are essential performance parameters where cylindrical parts are subjected to high internal and external loads, and a small error causes excessive deformation and results in failure of components.

### 3.2. An Optimization Methodology for Single and Multi-Responses

**Figure 3:**
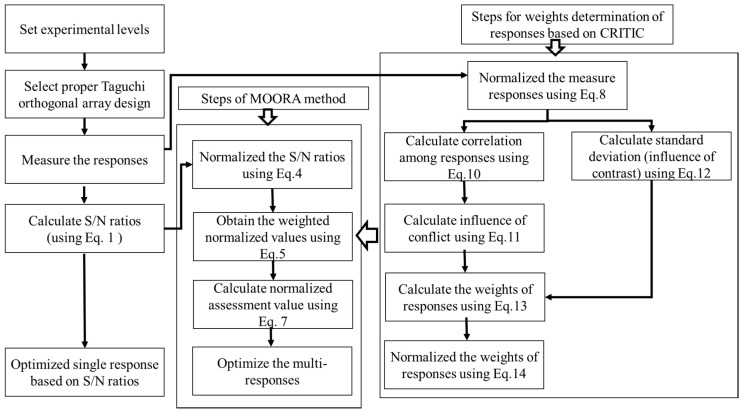
An optimization methodology for single and multi-responses. MOORA: multi-objective optimization on the basis of ratio analysis; CRITIC: criteria importance through inter-criteria correlation; S/N: Taguchi based signal to noise ratios.

Figure 3 shows the steps followed for proposed multi-responses optimization using the Taguchi based signal to noise (S/N) ratios, coupled with MOORA and CRITIC. The Taguchi based signal to noise (S/N) ratios determine the deviation in the quality characteristics of responses from desired values. The ratio of mean values (signal) and standard deviation (noise) gives an objective function to responses. If the desired response’s objective function is to minimize, then the smaller-the-better quality characteristics are measured using Equation (1). However, for maximization, the larger-the better quality is measured using Equation (2).

(1) SNratio=η=−10×log10(1n∑i=1nyi2)

(2) SNratio=η=−10×log10(1n∑i=1n1yi2)

where $y_{i}$ is the observed experimental value of response for experiment *i*, and *n* is the number of experiments.

The steps followed for MOORA in the present study are adapted from the study of Yusuf and Sebla [23] as follows:

**Step 1**: The decision matrix of order *m* × *n* is obtained based on the *S/N ratios* of measured responses. Rows show experimental runs, while the columns show the number of responses as expressed in Equation (3).

(3) $$D_{\left(S/N\right)}=\left[\begin{array}{cccc} \eta_{11} & \eta_{12} & \ldots & \eta_{1n}\\ \eta_{21} & \eta_{22} & \ldots & \eta_{2n}\\ \vdots & \vdots & \ddots & \vdots \\ \eta_{m1} & \eta_{m2} & \ldots & \eta_{mn} \end{array}\right]$$

**Step 2:** Normalized *S/N ratio* values using Equation (4).

(4) $$\eta_{ij}^{\prime }=\frac{\eta_{ij}}{\sqrt{\sum_{i=1}^{m}\eta_{ij}^{2}}}j=1,2,\ldots ,n$$

where $\eta_{ij}^{\prime }$ is the dimensionless number. As the *S/N ratio* values can be negative or positive, so the interval of normalized values lies in the range [−1, 1].

**Step 3:** Calculate the weighted normalized decision matrix of *S/N ratios* values using Equation (5).

(5) $$x_{ij}^{}=\eta_{ij}^{\prime }\times w_{j}\;j=1,2,\ldots ,n$$

where $w_{j}$ is the weight of jth response and $\overset{n}{\underset{j=1}{\sum }}w_{j}=1$.

**Step 4:** For multi-response optimization, weighted normalized assessment values were calculated. If the problem involves conflicting objective functions i.e., maximization and minimization, weighted normalized assessment values can be calculated using Equation (6). If the responses have similar objective functions i.e., either maximization or minimization, Equation (6) can be reduced to Equation (7).

(6) $$z_{i}=\sum_{j=1}^{g}x_{ij}^{}-\sum_{j=g+1}^{n-g}x_{ij}^{}\;j=1,2,\ldots ,n$$

(7) $$z_{i}=\sum_{j=1}^{n}x_{ij}^{}$$

where, $z_{i}$ is the weighted normalized assessment values, *g* is the number of responses to be maximized, (*n*−*g*) is the number of responses to be minimized.

**Step 5:** The experimental runs are ranked based on the highest $z_{i}$ value.

**Step 6:** Finally, optimal levels are obtained by computing the average values of $z_{i}$ for each factor at each level. The higher average value $z_{i}$ corresponds to the optimal level for an individual factor.

The procedure used to determine the weights of responses using CRITIC is as follows:

**Step 1:** The measured response was normalized, depending upon the objective function. For minimization (cost criteria) of objective function use Equation (8), while for maximization (benefit criteria) use Equation (9).

(8) $$\delta_{ij}=\frac{y_{ij}-\min(y_{j})}{\max(y_{j})-\min(y_{j})},\;j=1,2,\ldots ,n,\;i=1,2,\ldots ,m$$

(9) $$\delta_{ij}=\frac{\max(y_{j})-y_{ij}}{\max(y_{j})-\min(y_{j})}$$

**Step 2:** Determine the correlation between the responses using Equation (10).

(10) $$\rho_{jk}=\frac{\sum_{i=1}^{m}\left(\delta_{ij}-\bar{\delta_{j}}\right)(\delta_{ik}-\bar{\delta_{k}})}{\sqrt{\sum_{i=1}^{m}{\left(\delta_{ij}-\bar{\delta_{j}}\right)}^{2}\sum_{i=1}^{m}{\left(\delta_{ik}-\bar{\delta_{k}}\right)}^{2}}},\;j=1,2,\ldots ,n,\;i=1,2,\ldots ,m$$

**Step 3:** Calculate the degree of conflict between responses using Equation (11).

(11) $$\lambda_{j}=\sum_{k=1}^{m}\left(1-\rho_{jk}\right)$$

**Step 4:** Compute the standard deviation showing degree of contrast between responses using Equation (12).

(12) $$\sigma_{j}=\sqrt{\frac{\sum_{i=1}^{m}{\left(\delta_{ij}-{\bar{\delta }}_{j}\right)}^{2}}{m}},\;j=1,2,\ldots ,n,\;i=1,2,\ldots ,m$$

**Step 5:** Calculate the amount of information emitted by combining both degree of conflict and contrast using Equation (13). This shows the relative importance of responses (weights) and higher values of $\psi_{j}$ represent higher importance.

(13) $$\psi_{j}=\lambda_{j}\sigma_{j}$$

**Step 6:** Finally, the normalized weights of responses are obtained by applying Equation (14).

(14) $$w_{j}=\frac{\psi_{j}}{\sum_{k=1}^{m}\psi_{k}}$$

## 4. Results and Discussions

### 4.1. Probability Plots and Analysis of Variance (ANOVA)

**Figure 4:**
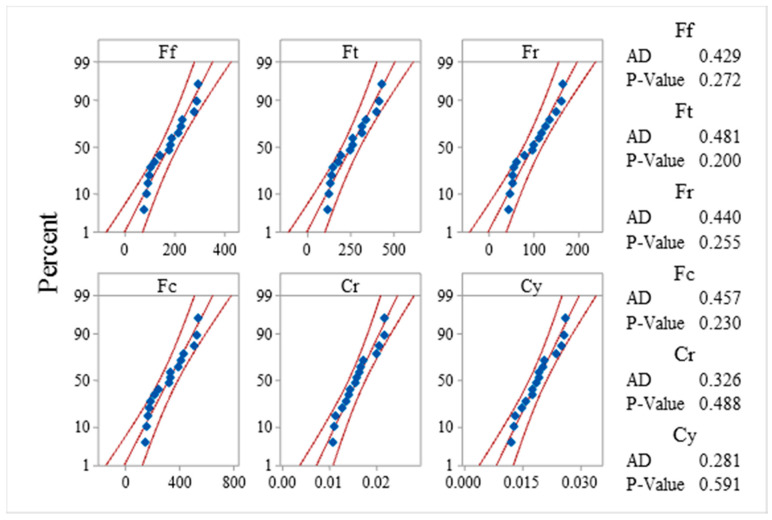
Probability plots for responses. AD: Anderson Darling test.

The distribution of experimental data was analyzed based on the probability plot. The plots are plotted at a 95% confidence interval and are shown in Figure 4. This shows that data points for all measured responses fall near the middle fitted line, and it is assumed that data follow normal distributions. The Anderson Darling (AD) test statistics value and *p*-value decide the null hypothesis’s acceptance and rejection concerning the normal distribution of data. The AD test statics value for each response data being low and the *p*-value being higher than 0.05, justifies that the collected data are normally distributed and can be used further for experimental analysis and optimization.

Analysis of variance (ANOVA) was performed at a 95% confidence interval, to analyze the effect of the cutting process parameters on responses. Table 3 shows the ANOVA results for the components of cutting forces. It shows that the feed rate has a significant effect on three components of cutting force namely F_f_, F_t_, F_r_ and F_c_ as their *p*-values are less than 0.05; however, the depth of cut is found to be significant for only F_f_ and F_r_. The cutting speed, positive rake, and cutting conditions are found to be insignificant. The average percentage contribution of feed rate for F_f_, F_t_, F_r_ and F_c_ is higher i.e., approximately 50%, followed by the depth of cut which is approximately 34%. The average percentage contribution for cutting speed, positive rake angle, and cutting conditions are relatively low i.e., approximately 7%, 3.5%, and 3%. These results are in line with the data available in the literature. For example, Kannan et al. [11]; Reis and Abrao [19]; Sood et al. [40]; concluded that feed rate has a higher contribution towards cutting forces in the turning of aluminum alloys and its composite matrix. Agustina et al. [17] observed that for the aluminum alloy A97075, the most significant cutting parameter is feed rate, followed by the depth of cut and spindle speed. Similarly, studies related to the turning of other materials such as AISI 4340 steel [41], AISI 4140 steel [42], red brass [43], Inconel 718 [44], also showed that feed rate and depth of cut have a significant effect on cutting forces while cutting speed is found to be insignificant.

Table 4 shows the ANOVA results for shape deviations of bars in terms of circularity and cylindricity deviation. The results show that feed rate is the most significant factor for both circularity and cylindricity deviation, while the spindle speed, depth of cut, positive rake angle, and cutting conditions are found to be insignificant. For the circularity deviation, feed rate has a higher percentage contribution of 49.02%, followed by the depth of cut with 21.57% and spindle speed 22.06%, while positive rake angle and cutting conditions are the least contributing factors with percentage contributions of 3.92% and 2.45%, respectively. Similarly, for cylindricity deviation, the percentage contributions are; feed rate (47.42%), followed by spindle speed (22.26%), depth of cut (20.32%) positive rake angle (4.84%) and cutting conditions with least contribution of 3.23%. These findings are supported by literature. For example, Patel et al. [8] studied that in the turning of the aluminum alloy Al 7075, feed rate had a significant effect on circularity and cylindricity error, followed by the depth of cut, while the cutting speed is insignificant. Rafai and Islam [45] concluded that feed rate, followed by cutting speed, and depth cut were influencing factors for diameter error and circularity in the dry turning of AISI 4340. Cui and Han [46] analyzed that the cutting parameters affecting shape error are depth of cut and feed rate, while the spindle speed has an insignificant effect in the turning of C45 steel.

### 4.2. Optimization Based on S/N Ratios for Individual Responses

The individual responses are optimized based on the Taguchi-based signal-to-noise ratio (S/N) ratio analysis. The primary objective function for cutting forces (F_f_, F_t_, F_r_, and F_c_) and shape deviations (C_r_ and C_y_) are similar i.e., minimization. So the smaller-the-better quality characteristic function is applied to all responses, using Equation (1). Independently from the quality characteristics, higher values of S/N ratios represent the best performance of responses corresponding to the desired experimental run.

**Figure 5:**
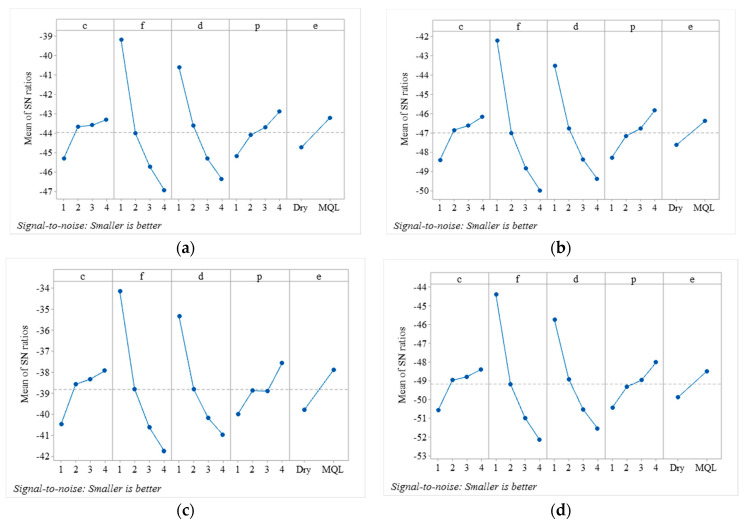
Main effect plot for means of Signal to noise ratio of components of cutting forces (**a**) feed force (**b**) tangential force (**c**) radial force (**d**) resultant force.

**Figure 6:**
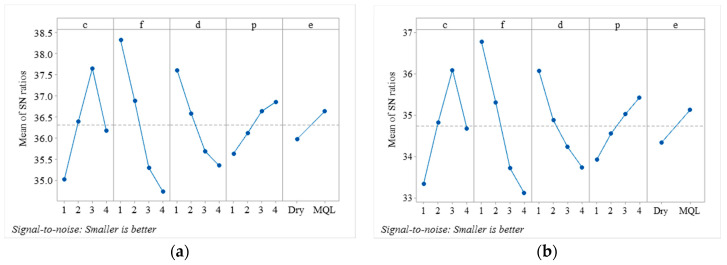
Main effect plot for means of Signal to noise ratio of shape deviations (**a**) circularity (**b**) cylindricity.

Table 5 shows the S/N ratios calculated for individual responses. The results show that the higher S/N ratios were obtained for the components of cutting forces (i.e., F_f_, F_t_, and F_c_) at experimental run five (having *c* and *d* at level two, *f* at level one, *p*, at level three and cutting condition at level two i.e., MQL). However, for F_r_, higher S/N ratios were observed in experimental run six (having *c* and *f* at level two, *d* at level one, *p*, at level four, and *e*, at level two i.e., MQL). For C_r_ and C_y_, the higher S/N ratios computed are found in experiment nine (*c* and *d* at level three, *f* and *e*, at level one (dry), and *p*, at level four. The mean values of S/N ratios are computed at each level for each factor, and higher mean values of S/N ratios correspond to the optimal level. Figure 5a–d illustrates the main effect plot of mean values of S/N ratios for components of cutting forces (F_f_, F_t_, F_r_ and F_c)_, respectively). It shows that the final optimal levels based on mean S/N ratios of all components of cutting forces are *c* and *p*, at level four (800 m/min and 25°), *f* and *d* at level one (0.1 mm/rev and 1 mm) and *e*, at level two (MQL). Figure 6a,b depicts the mean S/N ratios of C_r_ and C_y_, respectively. The optimal levels obtained are *c*, at level 3 (600 m/min), *f* and *d* at level one (0.1 mm/rev and 1 mm), *p*, at level four (25°) and *e,* at level two (MQL).

### 4.3. Main Effect Plots of Means of Cutting Forces

**Figure 7:**
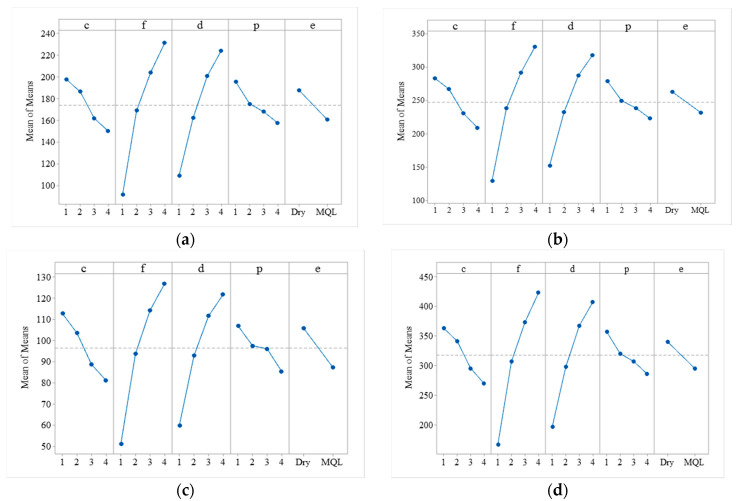
Main effect plot for means of components of cutting forces. (**a**) feed force (**b**) tangential force (**c**) radial force (**d**) resultant force.

The effect of the cutting process parameters on individual responses is studied through the main effect plots of means. Figure 7 shows the main effect plot for means of a component of cutting forces i.e., F_f_, F_t_, F_r_, and F_c_. Figure 7a indicates that the feed forces (F_f_) decrease with an increase in cutting speed and positive rake angle from low level to high level (i.e., 200 m/min to 800 m/min, and 10° to 25°), however, it increases with an increase in the feed rate and depth of cut from a low level to a high level (i.e., 0.1 mm/rev to 0.4 mm/rev and 1 mm to 2.5 mm). It is also observed that with the application of MQL, the F_f_ decreases. Similar results are obtained for tangential force (F_t_), radial force (F_r_), and resultant force (F_c_), as illustrated in Figure 7b–d. These results are in good agreement with the literature. Kannan et al. [11] observed that under both dry and MQL conditions, cutting forces increase with an increase in feed rate. This is attributed to the increased contact area between the cutting tool and workpiece, which causes an increase in temperature at the interface and results in the formation of built-up edges. They also observed that with an increase in cutting speed the formation of built-up edges reduces, therefore reducing the cutting forces. High cutting speed results in high heat generation in the workpiece, which makes the workpiece more plastic and makes machining easier. According to Sood et al. [40], an increase in feed rate increase the thickness of chips and causes increases in the cutting force; the same findings are also supported by Ajay and Vinoth [9]. Sood et al. [40] also observed that with an increase in cutting speed, the extent of interaction between tool and chip decreases, and also the formation of built-up edges reduces, therefore, reducing the cutting forces. An increase in depth of cut increases the shear area, so higher tangential forces followed by feed force and radial force are required to cut the material [19]. Saleem et al. [47] investigated that an increase in the positive rake angle decreases the cutting forces. This is attributed to an increase in the tool and chip contact area resulting in the easy formation of chips. They also found that the chip–tool interface temperature increases with an increase in positive rake angle, resulting in an increase in strain in the workpiece and reduces the cutting forces. According to Dhar et al. [48], a positive rake angle causes tool edge sharpening. The tool sharpness reduces cutting forces and improves the surface quality of the machined specimen, but it reduces the tool life significantly. Additionally, an increase in cutting speed or feed rate damages the tool sharpness and causes an increase in the shape deviations of machined parts.

### 4.4. Main Effect Plots of Means of Shape Deviations

**Figure 8:**
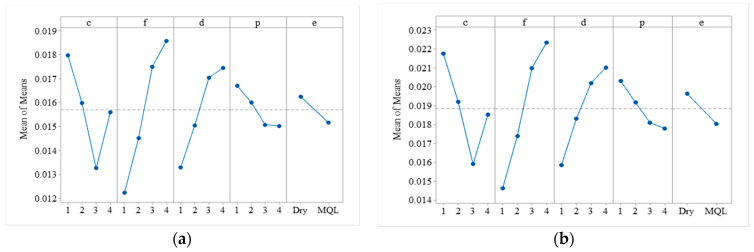
Main effect plot for means of shape deviations. (**a**) circularity (**b**) cylindricity.

Figure 8 shows the main effect plots of means of circularity and cylindricity. It shows that the mean values of both circularity and cylindricity decrease with an increase in the cutting speed from level one (400 m/min) to level three (600 m/min), but increase with further increases in cutting speed from level three (600 m/min) to level four (800 m/min). The circularity and cylindricity deviation increase with an increase in feed rate and depth of cut from a low to high level (i.e., 0.1 mm/rev to 0.4 mm/rev and 1–2.5 mm). However, with an increase in the positive rake angle, the circularity and cylindricity deviation decreases. Further, the plots also show that both circularity and cylindricity deviations decrease with the application of MQL. These results are in line with the literature. The possible reason for the increase in circularity and cylindricity deviation with an increase in cutting speed is spindle error and elastic deformation of the workpiece due to built-up edges, tool wear, and variation in cutting forces during turning of the aluminum alloy [8] and steel [45,46]. The other possible reason could be that the aluminum alloy has a low geometrical moment of inertia due to its structure, so during machining, at high cutting speed, this results in buckling and produces deflection forces which cause shape deviations. According to Sreejith [18] the increase may also occur due to the thermal softening of an aluminum alloy at high cutting speed. At lower feed rates, the cutting forces decrease because of small coefficient friction, and this results in minimum deviation during turning operation [49]. A higher positive rake angle reduces cutting forces, as evident from cutting forces analysis, so this may also be one reason for the shape deviation reductions in round bars.

### 4.5. Multi-Response Optimization

For multi-response optimization, the algorithm applied has been discussed in the optimization methodology section. First, the decision matrix of order 16 × 6 (experimental runs × number of responses) was obtained by calculating the signal to noise (S/N) ratios of individual responses, as tabulated in Table 5 using Equation (1). S/N ratios were normalized using Equation (4) to make the responses dimensionless. The normalized values are expressed in Table 6 and are in the range of [−1, 1]. Weighted normalized values are calculated using Equation (5) and are shown in Table 7. To obtain the best experimental run for combined multi-response, weighted normalized assessment values are calculated using Equation (7). The weighted normalized assessment values, along with the rankings, are shown in Table 8. It shows that the best combination of cutting process parameters and cutting conditions to minimize the responses simultaneously, is that in experimental run five (having a high weighted normalized assessment value of 0.089). It is ranked first, having a cutting speed and depth of cut at level two (600 m/min and 2 mm), feed rate at level one (0.1 mm/rev), positive rake angle at level three (20°) and cutting condition at level two (MQL). To obtain the final optimized levels, the average values of weighted normalized assessment values are calculated at each level for each factor, and larger average values correspond to the optimal level, as presented in Table 9. The optimized levels obtained are cutting speed at level three (600 m/min), feed rate and depth of cut at level one (0.1 mm/rev and 1mm), positive rake angle at level four (25°), and cutting condition at level two (MQL). Table 9 also illustrates, that based on the delta values, the feed rate is the more significant influencing factor followed by the depth of cut, cutting speed, positive rake angle, and cutting conditions.

The weights of responses in the present study are computed based on the CRTIC technique. First, the measured responses, as tabulated in Table 2, were normalized using Equation (8). The normalized values are shown in Table 10. Then, the correlation is obtained between each response based Equation (9) and these are tabulated in Table 11. The degree of conflict and contrast between responses was measured using Equations (10) and (11) and the results are depicted in Table 11. Relative importance of responses (weights) were obtained using Equation (12) and final normalized weights of individual responses were computed using Equation (13). The results show that C_r_ has a higher normalized weight of (0.324) and therefore has high priority followed by C_y_ (0.302), F_r_ (0.10), F_t_ (0.094), F_c_ (0.090), and F_f_ (0.090).

### 4.6. Comparative Study

Optimal levels, identified based on Taguchi S/N ratios coupled with MOORA and CRITIC are compared with other methods such as; Taguchi S/N ratios coupled with TOPSIS [49], Taguchi based grey relational analysis [50], composite desirability function [51], and S/N ratios coupled with grey relational analysis [52]. The initial control parameters set were *c*, *f*, *d*, and *p*, at level one. These were identified based on the worker experience and machine handbook. However, in practice, the optimal conditions are highly desirable. The optimal conditions identified based on the Taguchi S/N ratios coupled with MOORA and CRITIC are a *c* = 600 m/min, *f* = 0.1 mm/rev, *d* = 1 mm, *p* = 25° and the cutting condition is MQL. However, the optimal levels identified based on the other methods mentioned above are: a *c* = 400 m/min, *f* = 0.1 mm/rev, *d* = 1 mm, *p* = 25°, and an MQL cutting condition. Table 12 shows a comparison of responses obtained, based on initial control parameters and optimal parameters. It shows that the responses (i.e., cutting forces and shape deviations) at optimal levels identified based on the Taguchi S/N ratios coupled with MOORA and CRITIC performed better (i.e., lower components of cutting forces and shape deviations) compared to the initial control parameter settings and optimal levels identified based on other methods, such as the Taguchi grey relational analysis, composite desirability function, S/N ratios coupled with grey relational analysis, and Taguchi S/N ratios coupled with TOPSIS.

## 5. Conclusions

From the presented experimental and statistical study, following conclusions can be drawn regarding the turning of the 6026-T9 aluminum alloy under dry and MQL environments:

1. Based on ANOVA it is found that the feed rate is the most significant influencing factor for components of cutting forces (namely feed forces, tangential forces, radial forces, and resultant forces) and shape deviations (i.e., circularity and cylindricity). However, the depth of cut has a significant effect on the feed force and radial force, and an insignificant effect on tangential force, and shape deviations. Cutting speed, positive rake angle and cutting conditions are found to be have an insignificant effect on components of cutting forces and shape deviations.
2. The individual optimized levels identified, based on the Taguchi signal to noise (S/N) ratios for components of cutting forces are the same i.e., cutting speed and positive rake angle at a high level (800 m/min and 25°), feed rate and depth of cut at the low level (0.1 mm/rev and 1 mm) and cutting condition is MQL.
3. The optimized levels for circularity and cylindricity deviation are also similar i.e., the cutting speed at level three (600 m/min), feed rate and depth of cut at level one (0.1 mm/rev and 1 mm), positive rake angle at level four (25°) and the MQL cutting condition.
4. For multi-response optimization, the Taguchi based S/N ratio coupled with MOORA and CRITIC performed better compared to other multi-response optimization techniques such as the Taguchi grey relational analysis, composite desirability function, S/N ratios coupled with grey relational analysis, and Taguchi S/N ratios coupled with TOPSIS. The optimal parameters obtained based on the Taguchi S/N ratios coupled with MOORA and CRITIC are a cutting speed of 600 m/min, feed rate of 0.1 mm/rev, depth of cut of 1 mm, positive rake angle of 25° and the MQL cutting condition. The corresponding optimized responses obtained are tangential components of cutting forces: 113.55 N, feed force 60.28 N, radial force 18.51 N, a resultant component of three-component forces 129.9 N, circularity 0.0102 mm and cylindricity 0.0117 mm.
5. The turning operation in the MQL environment using vegetable oil can be considered more effective as compared to the dry environment in reducing the components of cutting forces and shape deviations of the machined work piece.
6. The detailed machining data generated related to aluminum alloys in the present study can be used as a benchmark permitting comparisons with other materials. It will help the industries to select the proper settings of cutting parameters for the cutting operation of aluminum alloy 6026–T9.
7. Future studies can incorporate the comparison of olive oil with other lubricants to analyze its performance. The effect of tool type, and tool nose radius on cutting forces and shape deviations also need to be explored. Further, the effect of cutting parameters on response variables such as tool chattering, fatigue strength, and energy consumption need to be investigated.

## Figures and Tables

**Table 1:** Cutting process parameters and experimental levels.

Process Parameters	Symbols	Levels			
		1	2	3	4
Cutting speed (m/min)	*c*	200	400	600	800
Feed rate (mm/rev)	*f*	0.1	0.2	0.3	0.4
Depth of cut (mm)	*d*	1	1.5	2	2.5
Positive rake angle (°)	*p*	10	15	20	25
Cutting conditions	*e*	Dry	MQL		

**Table 2:** Experimental runs based on Taguchi orthogonal array design L_16_ and measured responses.

Experimental Run	*c* (m/min)	*f* (mm/rev)	*d* (mm)	*p* (°)	*e*	F_f_ (N)	F_t_ (N)	F_r_ (N)	F_c_ (N)	C_r_ (mm)	C_y_ (mm)
1	1	1	1	1	1	96.23	135.60	55.60	175.33	0.0141	0.0175
2	1	2	2	2	1	184.90	258.70	111.30	336.90	0.0165	0.0202
3	1	3	3	3	2	232.00	337.60	135.34	431.41	0.0198	0.0235
4	1	4	4	4	2	278.20	401.80	148.40	510.75	0.0215	0.0258
5	2	1	2	3	2	75.25	113.50	45.20	143.49	0.0108	0.0131
6	2	2	1	4	2	83.17	117.30	43.15	150.13	0.0110	0.0127
7	2	3	4	1	1	290.90	411.20	161.00	528.80	0.0206	0.0251
8	2	4	3	2	1	295.70	425.90	164.40	543.93	0.0215	0.0259
9	3	1	3	4	1	92.05	125.50	51.20	163.84	0.0105	0.0121
10	3	2	4	3	1	225.20	316.70	125.07	408.24	0.0142	0.0174
11	3	3	1	2	2	117.60	170.30	62.00	216.05	0.0125	0.0148
12	3	4	2	1	2	212.70	310.40	116.00	393.76	0.0158	0.0193
13	4	1	4	2	2	102.20	143.10	52.20	183.43	0.0135	0.0158
14	4	2	3	1	2	182.50	260.10	95.20	331.70	0.0163	0.0193
15	4	3	2	4	1	176.12	245.80	98.40	317.99	0.0171	0.0206
16	4	4	1	3	1	138.60	185.90	78.40	244.78	0.0155	0.0184

**Table 3:** Analysis of variance for components of cutting forces.

	Source	DF	Adj SS	Adj MS	F-Value	*p*-Value	% Contribution
F_f_	*c*	3	5791	1930.5	3.82	0.214	6.63
	*f*	3	44,142	14,714.1	29.12	0.033 *	50.52
	*d*	3	30,377	10,125.8	20.04	0.048 *	34.77
	*p*	3	3128	1042.6	2.06	0.343	3.58
	*e*	1	2918	2918.2	5.77	0.138	3.34
	Error	2	1011	505.3			
	Total	15	87,368				
F_t_	*c*	3	13,819	4606	3.39	0.236	7.57
	*f*	3	91,649	30,550	22.47	0.043 *	50.20
	*d*	3	63,542	21,181	15.58	0.061	34.81
	*p*	3	6877	2292	1.69	0.393	3.77
	*e*	1	3944	3944	2.9	0.231	2.16
	Error	2	2719	1360			
	Total	15	182,550				
F_r_	*c*	3	2443.5	814.5	7.47	0.12	9.02
	*f*	3	13,217.9	4406	40.4	0.024 *	48.82
	*d*	3	8886	2962	27.16	0.036 *	32.82
	*p*	3	944.4	314.8	2.89	0.268	3.49
	*e*	1	1366.8	1366.8	12.53	0.071	5.05
	Error	2	218.1	109.1			
	Total	15	27,076.7				
F_c_	*c*	3	21,987	7329	3.76	0.217	7.41
	*f*	3	148,989	49,663	25.5	0.038 *	50.24
	*d*	3	102,720	34,240	17.58	0.054	34.64
	*p*	3	10,887	3629	1.86	0.368	3.67
	*e*	1	8060	8060	4.14	0.179	2.72
	Error	2	3896	1948			
	Total	15	296,537				

Degree of freedom (DF), Adjusted sum of square (Adj SS), Adjusted mean square (Adj MS), * significant.

**Table 4:** Analysis of variance for circularity and cylindricity deviations.

	Source	DF	Adj SS	Adj MS	F-Value	*p*-Value	% Contribution
C_r_	*c*	3	0.000045	0.000015	15.56	0.061	22.06
	*f*	3	0.0001	0.000033	34.57	0.028 *	49.02
	*d*	3	0.000044	0.000015	15.37	0.062	21.57
	*p*	3	0.000008	0.000003	2.68	0.284	3.92
	*e*	1	0.000005	0.000005	5.02	0.154	2.45
	Error	2	0.000002	0.000001			
	Total	15	0.000204				
C_y_	*c*	3	0.000069	0.000023	10.8	0.086	22.26
	*f*	3	0.000147	0.000049	22.94	0.042 *	47.42
	*d*	3	0.000063	0.000021	9.88	0.093	20.32
	*p*	3	0.000015	0.000005	2.41	0.307	4.84
	*e*	1	0.00001	0.00001	4.86	0.158	3.23
	Error	2	0.000004	0.000002			
	Total	15	0.00031				

Degree of freedom (DF), Adjusted sum of square (Adj SS), Adjusted mean square (Adj MS), * significant.

**Table 5:** Signal to noise ratio values of measured responses.

Experimental Run	F_f_	F_t_	F_r_	F_c_	C_r_	C_y_
1	−39.666	−42.645	−34.901	−44.877	37.016	35.139
2	−45.339	−48.256	−40.930	−50.550	35.650	33.893
3	−47.310	−50.568	−42.629	−52.698	34.067	32.579
4	−48.887	−52.080	−43.429	−54.164	33.351	31.768
5	−37.530 *	−41.100 *	−33.103	−43.136 *	39.332	37.655
6	−38.399	−41.386	−32.700 *	−43.529	39.172	37.924
7	−49.275	−52.281	−44.137	−54.466	33.723	32.007
8	−49.417	−52.586	−44.318	−54.711	33.351	31.734
9	−39.280	−41.973	−34.185	−44.288	39.576 *	38.344 *
10	−47.051	−50.013	−41.943	−52.218	36.954	35.189
11	−41.408	−44.624	−35.848	−46.691	38.062	36.595
12	−46.555	−49.838	−41.289	−51.905	36.027	34.289
13	−40.189	−43.113	−34.353	−45.269	37.393	36.027
14	−45.225	−48.303	−39.573	−50.415	35.756	34.289
15	−44.916	−47.812	−39.860	−50.048	35.340	33.723
16	−42.835	−45.386	−37.886	−47.776	36.193	34.704

* higher S/N ratios.

**Table 6:** Normalization of signal to noise ratios of responses.

Experimental Run	F_f_	F_t_	F_r_	F_c_	C_r_	C_y_
1	−0.225	−0.226	−0.224	−0.227	0.254	0.252
2	−0.257	−0.256	−0.262	−0.256	0.245	0.243
3	−0.268	−0.268	−0.273	−0.267	0.234	0.234
4	−0.277	−0.276	−0.278	−0.274	0.229	0.228
5	−0.213	−0.218	−0.212	−0.219	0.270	0.270
6	−0.218	−0.219	−0.210	−0.221	0.269	0.272
7	−0.279	−0.277	−0.283	−0.276	0.232	0.230
8	−0.280	−0.279	−0.284	−0.277	0.229	0.228
9	−0.223	−0.222	−0.219	−0.224	0.272	0.275
10	−0.267	−0.265	−0.269	−0.265	0.254	0.253
11	−0.235	−0.237	−0.230	−0.237	0.262	0.263
12	−0.264	−0.264	−0.265	−0.263	0.248	0.246
13	−0.228	−0.229	−0.220	−0.229	0.257	0.259
14	−0.256	−0.256	−0.254	−0.255	0.246	0.246
15	−0.254	−0.253	−0.255	−0.254	0.243	0.242
16	−0.243	−0.241	−0.243	−0.242	0.249	0.249

**Table 7:** Weighted normalized values of responses.

Experimental Run	F_f_	F_t_	F_r_	F_c_	C_r_	C_y_
1	−0.020	−0.021	−0.022	−0.021	0.082	0.076
2	−0.023	−0.024	−0.026	−0.023	0.079	0.073
3	−0.024	−0.025	−0.027	−0.024	0.076	0.071
4	−0.025	−0.026	−0.028	−0.025	0.074	0.069
5	−0.019	−0.021	−0.021	−0.020	0.088	0.082
6	−0.020	−0.021	−0.021	−0.020	0.087	0.082
7	−0.025	−0.026	−0.028	−0.025	0.075	0.069
8	−0.025	−0.026	−0.028	−0.025	0.074	0.069
9	−0.020	−0.021	−0.022	−0.020	0.088	0.083
10	−0.024	−0.025	−0.027	−0.024	0.082	0.076
11	−0.021	−0.022	−0.023	−0.021	0.085	0.079
12	−0.024	−0.025	−0.026	−0.024	0.080	0.074
13	−0.020	−0.022	−0.022	−0.021	0.083	0.078
14	−0.023	−0.024	−0.025	−0.023	0.080	0.074
15	−0.023	−0.024	−0.025	−0.023	0.079	0.073
16	−0.022	−0.023	−0.024	−0.022	0.081	0.075

**Table 8:** Normalized assessment values and ranking.

Experimental Run	Normalized Assessment Values	Rank
1	0.074	6
2	0.056	11
3	0.046	13
4	0.040	15
5	0.089	1
6	0.088	2
7	0.040	14
8	0.038	16
9	0.088	3
10	0.059	8
11	0.076	5
12	0.056	12
13	0.077	4
14	0.058	9
15	0.057	10
16	0.065	7

**Table 9:** Optimal levels based on average normalized assessment values and the importance of parameters.

Cutting Parameters	Levels				Optimal Levels	Delta	Parameters Rank
	1	2	3	4			
Cutting speed	0.054	0.064	0.070	0.064	3	0.016	3
Feed rate	0.082	0.066	0.055	0.050	1	0.032	1
Depth of cut	0.076	0.064	0.058	0.054	1	0.022	2
Positive rake angle	0.057	0.062	0.065	0.068	4	0.011	4
Cutting conditions	0.060	0.066			2	0.006	5

**Table 10:** Normalized values of measured responses based on CRITIC.

Experimental Run	F_f_	F_t_	F_r_	F_c_	C_r_	C_y_
1	0.095	0.071	0.103	0.080	0.327	0.391
2	0.497	0.465	0.562	0.483	0.545	0.587
3	0.711	0.717	0.760	0.719	0.845	0.826
4	0.921	0.923	0.868	0.917	1.000	0.993
5	0.000	0.000	0.017	0.000	0.027	0.072
6	0.036	0.012	0.000	0.017	0.045	0.043
7	0.978	0.953	0.972	0.962	0.918	0.942
8	1.000	1.000	1.000	1.000	1.000	1.000
9	0.076	0.038	0.066	0.051	0.000	0.000
10	0.680	0.650	0.676	0.661	0.336	0.384
11	0.192	0.182	0.155	0.181	0.182	0.196
12	0.623	0.630	0.601	0.625	0.482	0.522
13	0.122	0.095	0.075	0.100	0.273	0.268
14	0.487	0.469	0.429	0.470	0.527	0.522
15	0.458	0.423	0.456	0.436	0.600	0.616
16	0.287	0.232	0.291	0.253	0.455	0.457

**Table 11:** Correlation of responses and weights based on CRITIC.

Correlation						
	F_f_	F_t_	F_r_	F_c_	C_r_	C_y_
F_f_	1.000	0.999	0.995	1.000	0.917	0.921
F_t_	0.999	1.000	0.994	1.000	0.916	0.920
F_r_	0.995	0.994	1.000	0.995	0.918	0.925
F_c_	1.000	1.000	0.995	1.000	0.917	0.921
C_r_	0.917	0.916	0.918	0.917	1.000	0.997
C_y_	0.921	0.920	0.925	0.921	0.997	1.000
Other parameters of CRITIC						
Degree of conflict	0.090	0.092	0.098	0.089	0.333	0.316
Degree of contrast	0.335	0.342	0.339	0.340	0.325	0.319
Weights	0.030	0.032	0.033	0.030	0.108	0.101
Normalized weights	0.090	0.094	0.100	0.090	0.324	0.302
Priority	6	4	3	5	1	2

**Table 12:** Confirmation tests.

	*c*	*F*	*d*	*p*	*e*	F_f_	F_t_	F_r_	F_c_	C_r_	C_y_
Initial control parameters	400	0.1	1	10°	MQL	90.23	130.55	22.75	160.32	0.0141	0.0171
Taguchi S/N ratios and TOPSIS	400	0.1	1	25°	MQL	64.15	116.21	21.22	134.43	0.0111	0.0121
Taguchi based Grey relational analysis											
S/N ratios and Grey relational analysis											
Composite desirability function											
Taguchi S/N ratios, MOORA and CRITIC	600	0.1	1	25°	MQL	60.28	113.55	18.51	129.9	0.0102	0.0117
